# Responsible Innovation and De Jure Standardisation: An In-Depth Exploration of Moral Motives, Barriers, and Facilitators

**DOI:** 10.1007/s11948-022-00415-z

**Published:** 2022-12-07

**Authors:** Martijn Wiarda, Geerten van de Kaa, Neelke Doorn, Emad Yaghmaei

**Affiliations:** Faculty of Technology, Policy, and Management, Department of Values, Technology, and Innovation, Delft University of Technology, Jaffalaan 5, 2628 BX Delft, the Netherlands

**Keywords:** Standardisation, Responsible innovation, Standards

## Abstract

Standardisation is increasingly seen as a means to insert ethics in innovation processes. We examine the institutionalisation of responsible innovation in de jure standardisation as this is an important but unexplored research area. In de jure standardisation, stakeholders collaborate in committees to develop standards. We adopt the anticipation, inclusion, reflexivity, and responsiveness responsible innovation framework as our theoretical lens. Our study suggests that responsible standardisation processes should embody forms of these four dimensions. We investigate the institutionalisation of these dimensions and identify 96 factors that can motivate, hinder, or facilitate responsible standardisation. Factors were found through in-depth interviews with managers of a standard developing organisation. These are subsequently validated/rejected using surveys completed by committee representatives. The results suggest that the social desirability of standards is not self-evident. This study could pave the way for future research on responsible standardisation processes, complementing research on legitimacy, responsible innovation, and standardisation.

## Introduction

Responsible innovation (RI) has become a burgeoning research field in the past decades (Burget et al., 2016; Owen et al., 2012). It is often described as an inclusive and risk-mitigating approach to research and innovation and stems from long traditions of science and technology studies and ethics of technology (Owen & Pansera, 2019; Wiarda et al., 2021). Various scholars have emphasised the role of standards in implementing RI (e.g. Stilgoe et al., 2013; Wickson & Forsberg, 2015).

Standards have substantial technical and socio-economic impacts (Cowan, 1992) and affect many aspects of life (Timmermans & Epstein, 2010). Given this impact and ubiquity, it is important that standards are well-aligned with society’s values (Friedman, 1996; Ligtvoet et al., 2015; Wickson & Forsberg, 2015). This is particularly important in light of the voluntary nature of many standards as their use largely depends on societal support (Brunsson et al., 2012).

A large proportion of standards are developed through a process called de jure standardisation (Narayanan & Chen, 2012). In this process, stakeholders cooperatively create standards in committees that are only diffused when consensus is established (Wiegmann et al., 2017). Standard developing organisations (SDOs) facilitate this process (Simcoe, 2012).

Due to standards’ normative nature, standardisation is also increasingly seen as a means to proactively insert ethics in innovation processes (Busch, 2012; P. B. Thompson, 2021). While this potential of standardisation has been recognized in the standardisation and the RI literature, insight into how ethics-inspired frameworks fit existing standardisation practices is currently lacking (Inigo et al., 2021; Van De Kaa, 2013; Wickson & Forsberg, 2015).

Within in the field of RI, there is a consensus that responsible processes require at least forms of *anticipation*, *inclusion*, *reflexivity*, and *responsiveness* (Fraaije & Flipse, 2020; Stilgoe et al., 2013). This focus leaves room for a contextualised substantiation of RI. Actors can include diverse values and worldviews that form preconditions to understand what risks and uncertainties are ethically acceptable, challenge (implicit) drivers of researchers and innovators, and align innovations with society’s expectations.

However, it remains unclear how these four procedural dimensions are institutionalised in the process of de jure standardisation and what factors motivate, obstruct, or facilitate the uptake of these dimensions in this process.

This study addresses these two knowledge gaps by examining the extent to which the four dimensions of RI are institutionalised in the process of de jure standardisation. It subsequently explores what factors may affect their institutionalisation. In the remainder of this paper, we use the term ‘responsible standardisation’ to refer to standardisation processes that are shaped according to the four procedural RI dimensions.

We consider the Royal Dutch Normalisation Institute as our case study. By extension, this study derives a practical understanding of responsible standardisation and aims to lay the groundwork for future research on this topic.

This paper first positions the RI literature in the context of standardisation. It then explains the research design and proceeds with presenting and discussing the results.

## Four Dimensions of Responsible Innovation

Research on RI in the process of de jure standardisation is scarce, and an understanding of the concept of responsible standardisation appears absent. While this is the case, there is a general consensus that responsible processes in innovation should at least embody forms of *anticipation*, *inclusion*, *reflexivity* and *responsiveness* (AIRR framework; Burget et al., 2016; Fraaije & Flipse, 2020; Owen et al., 2021; Stilgoe et al., 2013), and processes that do so are expected to lead to more responsible outcomes (Fraaije & Flipse, 2020), i.e. responsible standards (Wickson & Forsberg, 2015).

First, anticipation urges actors to raise the question ‘what if…?’ (Ravetz, 1997) and to imagine what possible risks and uncertainties are present. Second, inclusion requires broad and early engagement with stakeholders to yield diverse insights (Bauer et al., 2021; Chesbrough, 2003). Third, reflexivity requires actors to challenge their drivers and align their work with their moral responsibility (Schuurbiers, 2011; Van de Poel & Zwart, 2010). Fourth, responsiveness calls for the capability to change the shape and direction of innovations in light of anticipatory, inclusive, and reflexive insights (Pellizzoni, 2004; Stilgoe et al., 2013).

The procedural nature of these four dimensions additionally helps cope with the so called Collingridge dilemma (Collingridge, 1980), i.e., the insufficiency of knowledge on how the shape innovations before path dependencies (David, 1994, 1995) and technological lock-ins emerge (Arthur, 1989). The AIRR framework consequently has the potential to support actors in proactively aligning research and innovation with early values and worldviews before this becomes problematic (Stilgoe et al., 2013). As a result, an enhanced ethical acceptability is suggested to lead to more legitimate and desirable outcomes that potentially yield more support and market acceptance (Fraaije & Flipse, 2020).

By departing from the normative view that these four procedural RI dimensions are necessary for responsible standardisation, this study attempts to bridge the literature of RI with standardisation, to provide a theoretical lens for understanding responsible de jure standardisation processes. The theory presented below is thus an aggregation of standardisation research that resonates with the four dimensions of RI.

### Anticipation and Standardisation

*Anticipation* requires actors to embrace the uncertain outcomes of standardisation. Imagination and systemic analyses can identify risks and benefits that innovations might bring about (Guston & Sarewitz, 2002; Stilgoe et al., 2013). De jure standardisation is an anticipatory process (Fomin, 2011) which predominantly occurs in the earliest phases of a technological development (David & Shurmer, 1996; Jakobs, 2006; Wiegmann et al., 2017). Uncertainties of future technologies, markets, and user values dominate this stage (David & Shurmer, 1996). Hence, foreseeing standardisation needs and outcomes is challenging (Featherston et al., 2016; Simcoe, 2005). Anticipatory standards are (1) characterised by their intention to guide future technological compatibility or interoperability; (2) created in (inter)national regulatory contexts that facilitate coordination between firms; and (3) openly accessible to all parties (Lyytinen & King, 2006). Their negotiations are particularly concerned with technical needs (Takahashi & Tojo, 1993). However, little research has evaluated whether anticipatory standardisation activities go beyond the purely technical and economic, and analyse and explore the broader societal impacts of standards.

### Inclusion and Standardisation

*Inclusion* refers to involving stakeholders throughout the development process to acquire diverse input. Inclusion is a core element of RI (Owen et al., 2021; Stilgoe et al., 2013) and collective action in standardisation (Hargrave & Van de Ven, 2006; Van den Ende et al., 2012). Stakeholder diversity provides for diverse information (De Vries, 1999; Markus et al., 2006). It is broadly acknowledged that this forms a requisite for novel knowledge (Allen, 1977; Arthur, 2007). As such, inclusion can shape and improve a standard’s content (Egyedi, 1996; Schmidt et al., 1998), and may lead to better and more ethical decision making (Nathan, 2015; Stahl, 2013). It does so by internalising society’s needs and values in standards (Evans et al., 1993; Friedman, 1996; Lundval, 1995; Markus et al., 2006). Inclusion can also increase the legitimacy of standardisation processes and outcomes (Forsberg, 2012; Fransen & Kolk, 2007; Lundval, 1995; Scharpf, 1999). Standard proposals that survive stakeholders’ scrutiny are expected to enjoy extensive support (Fischhoff, 2013).

The process should give stakeholders sufficient incentives to enter and stay in this process (Van de Kaa & De Bruijn, 2015). Generally, actors face numerous incentives (Blind & Mangelsdorf, 2016), but are often unable to participate due to insufficient resources even though they are affected by the standard (Hills, 2000; Jakobs, 2006). Unfortunately, this is frequently the case for the wider public (Forsberg, 2012; Timmermans & Epstein, 2010). As a result, a standard’s adoption can suffer from a lack of input as some needs are unmet (Foray, 1994). Standards need support from a critical mass and therefore naturally rely on a certain degree of inclusion. The marginalisation of population segments throughout standardisation is sometimes addressed by sponsoring ‘volunteers’ (Lehr, 1992) or by including various (e.g. user) coalitions (Foray, 1995; Hills, 2000; Markus et al., 2006). In the former, financial dependencies might influence the negotiation dynamics, whereas in the latter, resources can be merged to forge a more influential voice during negotiations.

### Reflexivity and Standardisation

*Reflexivity* refers to the ability of actors to apprehend how their activities, commitments, assumptions, and limited knowledge influence the development process. It considers how some perspectives might not be aligned with those of society (Fraaije & Flipse, 2020; Stilgoe et al., 2013). Reflexive standardisation requires detailed scrutiny of both this misalignment and the governance of standardisation processes itself. The literature on reflexivity distinguishes between first-order and second-order reflective learning. The first refers to the “consideration of problem definitions and evaluation of solutions” (Grin & Van De Graaf, 1996, p. 299) in light of the current value system and background theories (Van de Poel & Zwart, 2010). It is therefore concerned with improving standardisation based on the current notion of what responsible processes are. In second-order reflective learning, “value systems become the object of learning while in first-order learning these are taken for granted.” (Schuurbiers, 2011, p. 772). This meta-reflection can redefine the value system and challenge actors’ notion of responsibility. These two reflections hence consider the role and responsibility, respectively.

Standards are not merely the outcome of economic rationality but also the product of institutional values (Nickerson & zur Muehlen, 2006). Aligning values through reflective learning can be achieved by involving external stakeholders throughout the process and connecting and comparing their values with those of the committee. A lack of reflective learning in standardisation may be self-destructing (Hanseth et al., 2003). An example is the chosen human standard in biomedicine during the 1980s. White middle-aged males were the norm in biomedical experiments, while other groups were underrepresented. This consequently led to a resistance movement that disputed this practice (Timmermans & Epstein, 2010).

While inclusion enriches negotiations (De Bruijn & Ten Heuvelhof, 2008), it additionally complicates reflective learning by adding complexity (Hanseth et al., 2003). This suggests an inverse U-shaped relationship between inclusion and reflexivity: inclusion allows for reflective learning, but too much of it complicates and thus hampers this reflective learning.

### Responsiveness and Standardisation

*Responsiveness* means adequately reacting to insights acquired through anticipatory, inclusive, and reflexive activities (Stilgoe et al., 2013) to mitigate risks and seize opportunities (Pellizzoni, 2004). A responsive standardisation process continuously internalises input, demonstrates flexibility, and co-evolves with its changing environment. As such, standardisation is an iterative process of standard establishment and diffusion (Botzem & Dobusch, 2012). The frequency at which, and extent to which, a standard changes over time is captured by the notion of *standard flexibility* (Egyedi & Verwater-Lukszo, 2005; Van den Ende et al., 2012). This concept appears paradoxical as standards aim to provide compatibility through stability. However, flexibility can lead to a greater acceptance and stability of the standard in the long term (Van den Ende et al., 2012). Nevertheless, changing standards is made more challenging by network externalities (Callon, 1987), lock-in effects (Cowan, 1992), and standard complexity (Hanseth et al., 1996). Although responsiveness ought to be crucial for RI, there is a prevailing concern about too much flexibility (Egyedi, 1999; Hanseth et al., 1996). Some scholars solicit balance between stability and flexibility (Timmermans & Epstein, 2010), but it is unclear what exactly this entails. Along these lines, there is a tension between responsiveness and the stability of standards.

## Method

This study examines the extent to which RI is institutionalised in de jure standardisation. It does so by adopting the AIRR framework as its theoretical lens. Subsequently, it derives a practical understanding of responsible standardisation and the factors that might obstruct, facilitate, and motivate the four AIRR dimensions in the national de jure standardisation process.

As part of our method, the case of the Royal Dutch Normalisation Institute (NEN) was chosen to address the research aim of this study. NEN has been operational since 1916, and is a relatively large SDO. Its mission is to establish consensual, widely adopted, and socially desirable standards (NEN, 2021). The latter may imply that this SDO values and exhibits morally responsible process characteristics. Furthermore, this case's national character could accommodate for a relatively homogeneous (e.g. institutional) unit of analysis. Both these aspects contribute to a rich research environment.

**Fig. 1:**
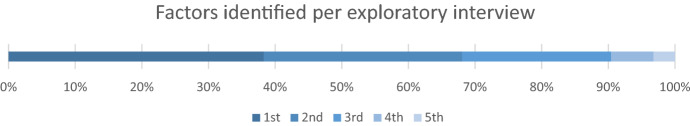
The proportion of factors identified per semi-structured interview, suggesting thematic saturation

This study deployed a mixed research method (Table 1). First, unstructured orientation interviews with various NEN employees (three consultants and two mid-level managers) were conducted to contextualize the study and gain familiarity with the SDO. This understanding allowed for the bridging of the academic concepts of RI to the practical context of standardisation. This is crucial as the implementation of RI is context-dependent (Jakobsen et al., 2019). Hereafter, in-depth semi-structured interviews with a large proportion of the management (one mid-level, and four high-level managers) were conducted to acquire qualitative insights on what responsible standardisation means to the SDO, how its current practices relate to the four dimensions of interest, and what factors motivate, hinder, or facilitate these. The interview questions can be found in Appendix I. The semi-structured interviews were transcribed and then analysed for any potential aspects that affect the four dimensions. The aspects (codes) were aggregated to factors (themes) affecting the four AIRR dimensions (categories). Data collection continued until thematic saturation was assumed (Fig. 1)—96 factors were identified after the last interview. All interviewees had extensive working experience at NEN, ranging from six to thirty years, with ample operational and management experience. Their perspectives are expected to be representative of the higher management.

**Table 1:** Overview of research process

Phase	Goal	Method	Length	Respondents	N
1	Orientation	Unstructured interviews	1 h	3 Consultants	5
				2 Mid-level managers	
2	Exploration	Semi-structured interviews	1–2.5 h	1 Mid-level manager	5
				4 High-level managers	
3	Validation	Survey	25 min	Consultants	28

Next, the factors identified through interviews have been validated/rejected by means of an anonymous survey sent via email. These were deployed among the SDO’s consultants (n≈100) of which roughly 1/3 responded. The consultants’ main task is to facilitate the standardisation process at the operational level with a chairperson's help. The consultants thus experience standardisation first-hand. They were asked to what extent they agreed with the factors by rating them on a 5-point Likert-scale.^1^ Other committee members could not be contacted due to privacy regulations. However, the nature of the consultants’ work provides them with a sufficient understanding of standardisation processes to validate or reject any process factor of interest. These mixed methods map the experience and perspective of both the ‘top- and bottom-layer’ of the organisation, which uncovers discrepancies. The results are described in the following section.

## Results

96 Factors were identified utilizing exploratory interviews (see Appendix II). An elaboration per factor, per dimension, is given below. These factors are referred to as lMn (motives), lBn (barriers), and lFn (facilitators), with l referring to an RI dimension (i.e. anticipation, inclusion, reflexivity, responsiveness) or responsible standardisation in general. n refers to the number of the respective factor. Appendix II indicates to what extent the consultants recognised the factors. This section first describes what responsible standardisation entails according to the SDO. Hereafter, the RI dimensions’ results are reported, followed by a summary of the survey results.

### Responsible Standardisation

According to the SDO, responsible standardisation is a process that establishes socially desirable standards. These standards make the world better, for example, by contributing to the environment, safety, and health. Suggested normative requirements for such a process are that all relevant stakeholders can participate, actively provide input, and that the process is transparent. All stakeholders should contribute to the standard’s development to create broad support. Respondents argued that standardisation strives to be responsible but that the role of an SDO is principally limited to neutrally facilitating the process while committee members determine the course and the outcome. This poses a dilemma. On the one hand, the SDO intends to be a neutral facilitator that only brings parties together and mediates between them. On the other hand, it recognizes that this might not be adequate for creating responsible standards.

Multiple motives to standardize responsibly were disclosed. Standards can affect technological developments and therefore substantially impact society. Furthermore, the SDO has an influential market position. Hence, the SDO’s role comes with a responsibility (SM1), which, according to the managers, NEN is also intrinsically motivated to meet (SM2). Respondents also indicated that committees are motivated towards responsibility out of their own interest (SM3) as members are affected by their standards.

Responsibility is instrumental to the organisation’s reputation as it provides credibility (SM4) in a continuously scrutinised environment. Socially desirable standardisation is believed to be inherently consensual and leads to increased market acceptance (SM5). When a consensual outcome is established collectively, then this assumedly leads to the best solution for the problem at hand (SM6). The international standardisation community considers standardisation as a tool for reaching the UN’s Sustainable Development Goals (SDGs; SM7). However, all committee members have their own agenda and reasons to initiate or be engaged in the standardisation process. The SDO’s influence on the outcome is therefore limited.

### Dimensions of Responsible Standardisation

#### Inclusion

##### Institutionalisation

Historically, industries established the SDO to facilitate agreements between industrial parties. Only around the 1990s did parties become aware of the value of including consumers. Although considerable effort is allocated to inclusion, all managers agreed that there was room for improvement. The average contemporary committee size was estimated to be ten to twelve stakeholders. These represent large groups of potentially affected actors. However, some standards are used by thousands of adopters. This poses the question of whether committees are inclusive enough. Respondents also mentioned that committee compositions should be more gender diverse and include “[economically] weak stakeholders” such as start-ups and activists.

##### Motives

Inclusion is a requisite for responsible standardisation (IM1). An inclusive committee represents society's interests to ensure the outcome is desirable and, hence, adopted (IM2). One of the managers stated: “If it is not supported by society, then there is no point in continuing the process at all”. Inclusive committees benefit from their members’ relevant know-how, resulting in better standards (IM3). This is claimed to be essential, as the SDO is not an expert on the content. An SDO is an expert in bringing parties together and mediating between them. If done well, the adopters of the standard are expected to feel as if they established the standard themselves, which causes them to perceive it as a logical solution (IM4). Inclusion is a primal need as stakeholders determine what a valuable standard is. It is not merely the standard that is valuable. The committee’s stakeholder network also provides value on itself (IM5), e.g., knowledge exchange.

##### Barriers

Various factors form barriers to inclusion. Stakeholders are not always aware that a standardisation process exists (IB1) or lack awareness of its importance (IB2). They can have difficulties in finding relevant standardisation processes and already established standards (IB3). They are not always able to be involved effectively (IB4), for example, due to power inequalities among committee members (IB5). Stakeholders are often unable to evaluate the benefits that parties have gained through involvement in standardisation (IB6). Other barriers include lack of time (IB7), priorities (IB8), or interest (IB9). A simple invitation from an SDO to the relevant stakeholders is often not enough. According to all managers, NEN’s business model is undoubtedly a barrier to inclusion (IB10). Stakeholders are required to pay a participation fee which impedes some stakeholders from joining. Subsequent expenditures (e.g., travel costs) increase this hurdle. Some stakeholders lack adequate knowledge (IB11) and some falsely assume their own shortcomings (IB12). The technical nature of standardisation (IB13) only adds to these last two barriers. Managers admitted that getting the last 20% of actors on board can take 80% of the effort. An SDO might also have difficulties understanding the role of stakeholders (IB14). A philanthropic organisation, for example, can be an extension of an industrial party. It is then not always clear whether this party acts on behalf of the charity or the industry. Occasionally, committee members might show resistance to more, or specific, new members (IB15). If the committee’s composition is inadequate, a less formal standard type can be chosen that does not require full stakeholder representation or consensus. The type of standard thus corresponds with the degree of inclusion (IB16). Alternatives to formal standards are national practice guidelines (NPR), national technical agreements (NTA), and the fast track standard (NEN-spec).

##### Facilitators

Various facilitating factors could increase the inclusion of standardisation. A prevalent factor is financial support for weak stakeholders (IF1).’Stronger’ stakeholders and the government could provide this support. However, one of the managers stated: “The simple assumption is that we should open up and allow everyone to freely take part in it [standardisation]. You’ll remove barriers… but it’s definitely not the complete solution”. Another possible factor is managing expectations (IF2). Stakeholders must realise that standardisation should be an inclusive and consensual process rather than a one-party-centred-service. Besides, technology could play a facilitating role (IF3). The COVID-19 pandemic has shown that virtual meetings cost less, take place more frequently, and increase the participation of weaker stakeholders. It is unclear whether an entirely virtual process would be optimal as some interaction in person sparks a necessary mutual understanding. Moreover, inclusion by membership is not the only solution. Other forms of participation, such as public consultations, could increase engagement without demanding total commitment (IF4).

#### Anticipation

##### Institutionalisation

The common assumption is that if processes are inclusive enough and all interests are represented, then negative impacts will be anticipated and mitigated. However, not all standardisation processes are inclusive enough to do so, nor are all actors always able to anticipate the effects on behalf of their party (competently and effectively). The international context is claimed to be different. ISO standardisation must describe how the standard relates to SDGs and other challenges. This is difficult because the link between a particular, esoteric, and technical standard and its broader technological, economic, societal, and environmental impact is not self-evident. “Even when you are standardising a bolt, you’ll have to elaborate on the economic and social impact. Often these questions are utterly difficult to answer”. Answering these inquiries in the national standardisation is not obligatory for committees. Here, ultimately, it is a gut feeling associated with a specific topic that prompts a standard’s ethical questions. For example, Artificial Intelligence (AI) is perceived as morally alarming, whereas an ICT system might not evoke the same concerns. Although standardisation considers ‘simple use’ and ‘misuse’ of standards, no comprehensive anticipatory study is conducted.

##### Motives

Anticipation is motivated by the belief that it is necessary to create socially desirable standards (AM1), increase market adoption (AM2), and ensure quality (AM3). It could also prolong the relevance of standards (AM4). Although committee members could be intrinsically motivated to anticipate the impacts (AM5), it is of paramount importance that an SDO determines and agrees on the role that it intends to fulfil. Does it merely intend to be a neutral facilitator/mediator and rely on the committees' willingness? Or will it stimulate the committee to conduct anticipatory activities? This is a topic of discussion for the SDO.

##### Barriers

Generally, anticipation is voluntary and thus requires willingness (AB1), which depends on the composition of the committees (AB2). Members may lack technical knowledge (AB3), financial resources (AB4), and anticipatory skills (AB5), which can obstruct anticipation. Moreover, members are not always aware of the current state of affairs (AB6) and the likely outcome of the process (AB7). The nature of standards (AB8) and their versatile use can hinder anticipation (AB9). As an example of the latter, standardising geographic maps might not have an apparent controversial impact, but these standards are essential for warfare missile systems. The standard’s use might only become apparent in hindsight. Even if members are willing to anticipate, their capacity can suffer from a lack or superficiality of anticipatory tools (AB10).

##### Facilitators

First, inclusion could increase the committee’s anticipatory capacity (AF1). Second, managers mentioned that technology could increase the process’ transparency so that public scrutiny can hold committees more accountable for their actions (AF2). This might incite anticipation. A fully transparent process is challenging to achieve as committee members are frequently inclined to disclose sensitive or classified information pertinent to the standard. Maintaining information asymmetries is namely crucial for the competitive advantage of many members.

#### Reflexivity

##### Institutionalisation

Reflective learning is done both proactively and reactively, and both in the first-order and second-order. The SDO’s decisions predominantly relate to including stakeholders and establishing consensus. Therefore, these aspects undergo most first-order reflection. A managers stated: “We continuously assess the composition of the standardisation committee. For this, we use a stakeholder analysis. We consider which societal aspects are relevant, which parties relate to these aspects, and how we could involve them”. Moreover, committees are requested to reflect on their completeness and to provide feedback on the SDO’s services. Employees are trained and assessed on their performance through an internal academy, a complementary mentor, and a monitoring policy committee. The members are also guided by a consultant, organisational statutes, codes of conduct, and regulations. The latter three are considered safety nets. The SDO is a member of international organisations, which impose additional quality criteria, e.g. committee composition requirements. Standards always enjoy a period of public scrutiny before publication, and external events frequently trigger reflective discussions on how these relate to the committees. One manager provided an example: “The huge Schiphol Airport fire was, of course, horrible. It led to questions in our committee [fire safety] on what our role is in this. Could we have prevented this? Can this be solved with standards?” Although reflective mechanisms are in place, the committee’s reflective capacity is still partly dependent on its members’ willingness and initiatives.

##### Motives

The SDO and its committees reflect on their actions as this is assumed to be essential for increasing the social desirability (RM1) and market adoption of standards (RM2). This suggests that reflection is at least partly motivated instrumentally.

##### Barriers

Barriers to reflexivity are a lack of inclusion (RB1) and transparency (RB2), the ambiguous interpretation of codes of conduct (RB3), the hidden agenda of committee members (RB4), and the complexity of the standardisation process (RB5). Reflective learning is also more complicated at the organisational level than in a single committee as standardisation contexts differ in terms of sector and topic (RB6). It is hence hard to comprehend the generalisability of lessons learned in one committee to other committees. Likewise, reflecting on standards' impact is also difficult as an agreed upon definition or impact assessment is lacking (RB7).

##### Facilitators

Although misinterpretation of formal rules, guidelines, and processes can obstruct reflexivity, these mechanisms are principally established to enhance this (RF1). Evaluation tools (RF2), training (RF3), external controlling bodies such as ISO (RF4), and internal bodies such as a policy committee (RF5) can increase reflective capacity. Furthermore, technology can facilitate public scrutiny, stimulate inclusion and transparency, and hence incite reflective activities (RF6). External incidents can also trigger reflection (RF7). Perhaps more critical is the committees’ awareness of their moral obligation to society, and that their actions will affect everyone in the standard’s system (RF8).

#### Responsiveness

##### Institutionalisation

Reaching consensus is a lengthy process that can take three to four years. Some sectors require a faster pace than others considering differences in the rate of technological change. Processes can be cancelled if they exceed the predetermined time frame. A standard is re-evaluated every five years if no earlier request is made. All managers admitted that this is too long, but also necessary for attaining consensus and stakeholder support. If this is not required, then an NTA, NPR, and NEN-spec are quicker alternatives. Although these can provide a faster solution to problems, they are not likely to respond adequately to all stakeholders' needs and values and will therefore lack full support. An example of an alternative standard is the ‘non-medical facemask for public use’ (NEN-spec 1) established in a record time of three weeks as a response to COVID-19. This was partly possible because consensus was not (yet) a hard requirement. Rather, an accelerated agreement on the masks’ quality was prioritised. This presented disadvantages as some dimensions appeared incorrect. However, the NEN-spec can compensate for potential flaws by its six-month expiration period. The standard must then either be improved, terminated, or upgraded to e.g., a formal standard. An advantage is that committees do not have to start from scratch.

##### Motives

Initiating a standardisation process is motivated by the notion that society is better off when aspects are uniformly agreed upon (ResM1). Standards can also be a response to SDGs (ResM2). Yet, calls for standards are not automatically rejected for the simple reason that they are not beneficial for society. The committee on smoking is an example. Although its contribution to society may be questionable, the law ultimately confines the scope of the SDO’s activities.

Responsiveness in standardisation is furthermore essential because it requires a genuine appreciation and internalisation of every input to achieve consensus (ResM3), ensure quality (ResM4), and increase market acceptance (ResM5). Calls for formal standards are occasionally rejected based on the belief that consensus appears unattainable. If parties do not seek consensus, they should not establish a standard as it would limit its adoption. Once a standard is established, responsiveness is critical to ensure that the standard’s value is maintained (ResM6).

##### Barriers

Responsiveness may also mean to not do, or discontinue, something. But there are no clear requirements for when to do so (ResB1). One manager referred to the committee for sustainable proteins (e.g., peas, crickets). Although its work is beneficial for the environment, it can also be detrimental to the incumbent industry for conventional proteins (e.g., eggs, meat). It is not always evident what role an SDO should play in these transitions. No protocols are in place to cope with these market dynamics.

The time-to-consensus is dependent on the committee. Conflicting goals (ResB2) and hidden agendas of committee members (ResB3), the complexity of the standard (ResB4), last-minute participators (ResB5), emotionally involved parties (ResB6), and conflicting fundamental values (ResB7) are all factors that can slow down or hinder consensus. One of the managers gave an example: “It [the European standard committee for halal food] led to a discussion about the interpretation of the Quran. Seeing that this concerns the very fundamental life philosophies of individuals, we ultimately concluded that consensus was unattainable”. Some topics are therefore also less susceptible to consensus (ResB8).

Inclusion also poses a dilemma concerning responsiveness (ResB9). Too little inclusion might accelerate the process of consensus but result in possibly excluding important insights. It could induce a misleading belief that the achieved consensus emanates societal support. Excessive inclusion might provide all input, but negotiations presumably prolong and could exceed the predetermined time limit. The SDO struggles with this. When, despite efforts to include a wide range of actors, the diversity of parties is too limited, a possibility would be to terminate the process. However, this is not always an option because the national SDO does not enjoy full autonomy (ResB10). Being a member of the European Committee for Standardisation (CEN), for instance, comes with both benefits and dependencies. If a European standard is developed, it is by definition applicable in all member states. This, therefore, compels nations to participate.

After standards have been established, flexibility may become challenging. Responsiveness covers adjusting standards to changing needs, values, and environments. Nevertheless, not all parties are willing or able to adjust their innovations. Standards intend to provide stability, not constant change. Ergo the desire for stability (e.g., due to sunk investments) may hinder responsiveness (ResB11).

##### Facilitators

Factors that can shorten the time-to-consensus are the meeting mode (e.g. virtual; ResF1), the type of standard (ResF2), governmental pressure (ResF3), societal pressure (ResF4), the severity of the problem at hand (ResF5), the willingness of members to compromise (ResF6), group cohesion (ResF7), members’ realistic expectations of standardisation (ResF8), the frequency of meetings (ResF9), and a sense of urgency (ResF10). The facemask standard as a response to COVID-19 is a good example. It was established in record time as government pressure, the severity of the problem, and the sense of urgency influenced the willingness to compromise. As mentioned, the NEN-spec does not require complete consensus nor full stakeholder representation. The additional shift to virtual standardisation led to more effective and frequent meetings. Generally speaking, national standardisation affects responsiveness positively as it allows the national SDO to have more control in the process (ResF11). Finally, the sector type influences the time-to-consensus and the rate at which the standard needs to be improved (ResF12). The chairperson’s mediating competencies additionally affect the speed of standardisation (ResF13). The chair is not an SDO employee and is sometimes chosen based on technical expertise rather than coordinative skills. The mediator should be able to pinpoint the root cause of disagreements and “peel the layers of the onion one by one”. However, finding a willing candidate to take on this role is often challenging. The chair person is namely obliged to be neutral and invest more time.

### A Bottom-Up Perspective

**Fig. 2:**
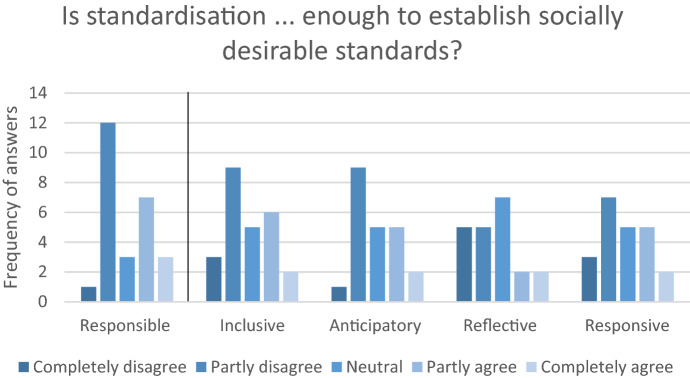
The consultant's perspective on whether standardisation is capable of establishing socially desirable standards

Consultants were asked whether de jure standardisation is, in their opinion, responsible enough to establish socially desirable standards. Notably, a large proportion disagreed that this is the case (see Fig. 2). Furthermore, they appeared sceptical on whether standardisation is inclusive and anticipatory enough. The results are less evident on the reflective and responsive capacity of the process.

Based on the survey’s median values for the 96 factors, none of the factors were categorically rejected. The medians indicate that consultants ‘completely agreed’ with the presence of 16 factors, ‘partly agreed’ with 58 factors, and were ‘neutral’ about 22 factors (see both Table 2 and Appendix II). No new factors were identified through the survey.

**Table 2:** Overview of the survey's results. To what extent do consultants recognise the presence of the identified factors

Dimension	Result survey (based on median)	Motives	Barriers	Facilitators
Responsible standardisation	Completely agree	2	N/A	N/A
	Partly agree	4	N/A	N/A
	Neutral	1	N/A	N/A
Inclusion	Completely agree	3	2	0
	Partly agree	2	7	4
	Neutral	0	7	0
Anticipation	Completely agree	3	0	0
	Partly agree	2	8	1
	Neutral	0	2	1
Reflexivity	Completely agree	0	0	1
	Partly agree	2	4	5
	Neutral	0	3	2
Responsiveness	Completely agree	1	0	4
	Partly agree	3	8	9
	Neutral	2	3	0
Total		25	44	27

## Discussion and Conclusion

This study examined the extent to which the procedural dimensions of RI are institutionalised in the process of de jure standardisation and identified 96 factors that might motivate, hinder, or facilitate these four dimensions (Appendix II). Sixteen of these form a set of most prevalent factors (Appendix III).

**Fig. 3:**
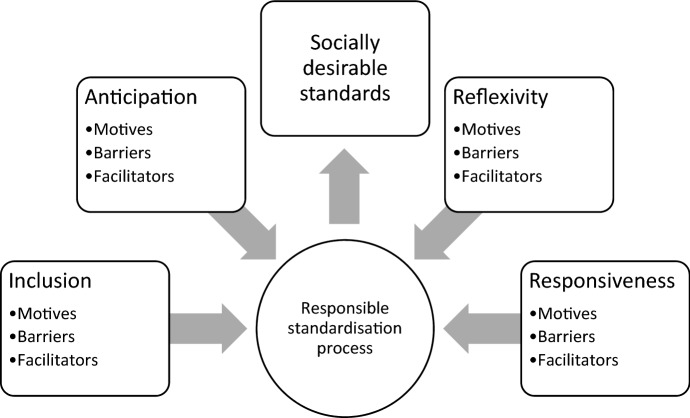
A conceptual framework for RI in the process of de jure standardisation

The SDO defines responsible standardisation as a process that establishes socially desirable standards. It recognises its moral obligation to society in facilitating such processes and deliberately reflects on re-shaping its organisation to accommodate this. This study shows that anticipation, inclusion, reflexivity, and responsiveness are motivated, and thus perceived to be necessary, to meet this obligation (Fig. 3). Besides, respondents believe that it could provide other significant benefits as well e.g. increased legitimacy, quality, and market acceptance of standards. However, RI’s extensive institutionalisation remains problematic for the following reasons.

First, the SDO tends to profile itself as a neutral facilitator. In contrast, RI might require to discard this division of moral labour and, for example, to proactively guide committees to engage in anticipatory activities. Therefore, there may be a tension between the SDO’s neutrality and RI. An additional tension is found in the committees as members have both obligations associated with their role and obligations towards society (Grinbaum & Groves, 2013).

Second, a large number of respondents indicated that standardisation might not be responsible enough to establish socially desirable standards. Aligning the role and societal responsibility can be difficult. If parties pursue the four dimensions, numerous factors might impede this effort. For example, our results suggest that transparency can incite reflexive and anticipatory behaviour. Yet, this seems unattainable as information asymmetries (e.g. Akerlof, 1970) remain important for the (sustained) competitive advantage of companies (Barney, 1991). Safeguarding this advantage is important for coopetition (Chiambaretto et al., 2019).

Third, respondents believe that if all stakeholders participate and represent their own interest, then all negative impacts will be mitigated in the process of reaching consensus. It is nevertheless unclear how this resolves, what is known in the literature as, the problem of many hands (D. Thompson, 2005), that is, the phenomenon that it is sometimes difficult to assign responsibility if a large number of people is involved in some activity. This may leave some important tasks unaddressed (Van de Poel et al., 2012). Hence, it seems unlikely that the AIRR framework suffices in adequately governing the standardisation process.

Fourth, the SDO and its committees are ill-equipped to meet the requisites of a responsible process. For instance, the lack of anticipatory skills and protocols leaves the committees empty handed. It is principally their gut feeling which indicates that emerging technologies such as AI require more attention than a simple bolt. How, and when, to anticipate remains therefore unclear. A clear definition of impacts is likewise absent. Although ‘hard impacts’ (quantitative) may come to mind, it is important not to overlook ‘soft impacts’ (qualitative; van der Burg, 2009). Identifying these might only become more difficult due to the versatility of a standard’s use and the novelty of future challenges that may arise. Like the deficiency of the other dimensions, the lack of anticipation makes responsiveness only more important as standards need agility in response to flaws that appear. From an evolutionary perspective, responsiveness is needed to accommodate for technological change, but allowing for flexibility is difficult as standards principally intend to provide stability (Van den Ende et al., 2012). Hence, a balance needs to be found between stability and flexibility.

Fifth, along the lines of the evolutionary economics tradition, we find that RI proves problematic in light of creative destruction (Schumpeter, 1934). RI requires a response to the interests and values of all parties. However, creative destruction inevitably changes the political order creating both ‘losers’ and ‘winners’. Following some scholars’ reasoning (Blok & Lemmens, 2015; de Hoop et al., 2016), standards that spur innovations that contribute to creative destruction could be regarded as irresponsible due to the negative impacts for the incumbent industry. This might need reconsideration, as creative destruction is generally perceived as necessary for long-term societal progress and wellbeing. In our case, the SDO had to take sides in the protein transition and refrain from neutrality. This confronts SDOs, and larger socio-technical systems, with a special moral lock-in (Bruijnis et al., 2015)—the conventional industry is unsustainable and considered unethical by some, but the alternative is also morally questionable.

While RI’s institutionalisation is influenced by these aforementioned aspects, we find that RI dimensions are also dependent on the type of standard and sector. This raises the question how different agreements and sectors relate to the four dimensions, and when particular types of agreements are legitimised. In our study, for instance, NEN-specs are found to be more responsive while formal standards are found to be more inclusive. Mapping the different properties of the de jure standardisation ‘toolkit’ in different sectors could provide valuable practical insights for innovation governance and policy and therefore seems a promising topic for future research.

This study has explored the institutionalisation of RI in de jure standardisation. Although many scholars often assume that standards are socially desirable, our results suggest that this is not self-evident. Still, we find ourselves at the beginning of RI’s institutionalisation in standardisation, and many research topics are left unexplored. As discussed above, this paper suggests that RI in practice occasionally proves itself problematic—prompting future research concerning concepts such as collective responsibility, foresight, market competition, and Schumpeterian patterns of innovation. Furthermore, this work complements existing research on moral legitimacy (Forsberg, 2012; Suchman, 1995), explicating the need for a better understanding of RI’s institutionalisation in the context of organisations (Owen et al., 2021) and innovation systems (Owen & Pansera, 2019).

## Appendix I: Interview Questions (Phase 2)

See Table 3

**Table 3:** An overview of the questions asked during the exploration phase

No	Question
*Preparatory*	
1	What is your position within NEN?
2	In what sector have you gained most standardisation experience?
3	How long have you worked for NEN?
*Part one*	
4	What does responsible standardisation mean to you as an employee of NEN?
5	What are reasons to standardise responsible?
6	What factors impede or obstruct responsible standardisation?
7	How can standardisation become more responsible?
8	Are you familiar with the terms ‘Responsible Research and Innovation’, ‘Responsible Innovation’, or ‘Maatschappelijk verantwoord innoveren’?^a^ If yes, could you please describe what these terms mean?
9	What is a successful standard according to you, and why?
*Part two*	
10	Inclusion in standardisation can be described as: “the active engagement of all stakeholders throughout the standardisation trajectory”. On a scale from 1 to 5, how inclusive do you think standardisation is? Could you please motivate your answer?
11	What motivates inclusion in the context of standardisation?
12	What impedes inclusion in the context of standardisation?
13	What facilitates inclusion in the context of standardisation?
14	Is standardisation inclusive enough to develop successful standards, and why (not)?
15	Anticipation in standardisation can be described as: “the process that aims to foresee potential societal, economic, and technological impacts in early phases of the standardisation process”. On a scale from 1 to 5, how anticipatory do you think standardisation is? Could you please motivate your answer?
16	What motivates anticipation in the context of standardisation?
17	What impedes anticipation in the context of standardisation?
18	What facilitates anticipation in the context of standardisation?
19	Is standardisation anticipatory enough to foresee important societal, economic and technological impacts of the standard, and why (not)?
20	Reflexivity in standardisation can be described as: “the continuous evaluation of whether the goal, activities, and outcomes of standardisation align with their moral obligation to society”. On a scale from 1 to 5, how reflexive do you think standardisation is? Could you please motivate your answer?
21	What motivates reflexivity in the context of standardisation?
22	What impedes reflexivity in the context of standardisation?
23	What facilitates reflexivity in the context of standardisation?
24	Is standardisation reflexive enough to develop socially desirable standards, and why (not)?
25	Responsiveness in standardisation can be described as: “a standardisation process that adequately changes standards based on the insights derived from the inclusive, anticipatory, and reflexive processes”. On a scale from 1 to 5, how responsive do you think standardisation is? Could you please motivate your answer?
26	What motivates responsiveness in the context of standardisation?
27	What impedes responsiveness in the context of standardisation?
28	What facilitates responsiveness in the context of standardisation?
29	Is standardisation responsive enough to develop socially desirable standards, and why (not)?
30	Are there any remaining comments, remarks, or side notes that you would like to share?

^a^ The Dutch translation for Responsible Innovation.

## Appendix II: An Overview of Identified Factors

See Table 4

**Table 4:** Overview of factors identified through interviews. Consultants indicated to which extent they recognized the factors by answering a survey: completely agree (1), partly agree (2), neutral (3), partly disagree (4), completely disagree (5)

Dimension	Type	No	Factor	Survey results^2^ Median ($\overline{{\varvec{x}} }$)
Responsible standardisation	Motive	SM1	SDO’s obligation to society	1 (1.41)
		SM2	Intrinsic motivation of SDO employees	2 (2.44)
		SM3	Out of committee members’ own interest	3 (2.46)
		SM4	To safeguard the credibility of SDO	1 (1.63)
		SM5	To increase market acceptance	2 (1.78)
		SM6	To ensure the quality of standards	2 (2.33)
		SM7	To reach the Sustainable Development Goals (SDGs)	2 (1.81)
Inclusion	Motive	IM1	To increase the responsibility of standards	1 (1.58)
		IM2	To increase the market adoption of standards	1 (1.58)
		IM3	To increase the quality of standards	2 (2.19)
		IM4	To establish a standard that is perceived as logical/self-evident	2.5 (2.50)
		IM5	To increase the value of the SDO’s stakeholder network	1 (1.58)
	Barrier	IB1	Unawareness of the process of standardisation	1 (1.62)
		IB2	Unawareness of the importance of participation in standardisation	2 (1.92)
		IB3	Difficulty of finding relevant standardisation processes	2 (2.24)
		IB4	Difficulty of being involved effectively	2 (2.04)
		IB5	The inequality in the influence which committee members have	3 (2.88)
		IB6	Lack of reflection on benefits a party might have gained through participation	2 (2.54)
		IB7	The limited time in which a standard needs to be developed	2 (2.12)
		IB8	A lack of a stakeholder’s priority	2 (2.20)
		IB9	A lack of a stakeholder’s interest	2 (2.33)
		IB10	The cost of involvement	1 (1.76)
		IB11	A lack of knowledge	3 (2.68)
		IB12	The stakeholder’s assumption that they are not competent enough	3 (2.92)
		IB13	The technical nature of some standardisation processes	3 (2.56)
		IB14	The ambiguous role a stakeholder might have	3 (2.82)
		IB15	Resistance from other committee members	3 (2.88)
		IB16	The type of standard (e.g. formal standard, NPR, NTA, NEN-spec, etc.)	3 (2.38)
	Facilitator	IF1	Financial support for economically weak stakeholders	2 (1.83)
		IF2	Management of stakeholders’ expectations	2 (2.13)
		IF3	Technology (e.g. virtual meetings, stakeholder feedback systems, etc.)	2 (2.09)
		IF4	Non-membership forms of participation (e.g. public consultations)	2 (1.87)
Anticipation	Motive	AM1	To increase the responsibility of standards	1 (1.77)
		AM2	To increase the market acceptance of standards	1 (1.78)
		AM3	To increase the quality of standards	2 (2.04)
		AM4	To prolong the potential relevance of standards	1.5 (2.05)
		AM5	Intrinsic motivation of committee members	2 (2.55)
	Barrier	AB1	A lack of willingness to anticipate	2 (2.04)
		AB2	The composition of the committee	2 (1.91)
		AB3	A lack of technical knowledge	2 (2.39)
		AB4	A lack of financial resources	2 (2.30)
		AB5	A lack of skills to anticipate	2 (2.68)
		AB6	Unawareness of the current state of affairs	3 (2.68)
		AB7	Uncertainty of the outcome of standardisation	3 (2.65)
		AB8	The technical nature of some standards	2 (2.39)
		AB9	Uncertainty of how the standard will be used	2 (2.73)
		AB10	A lack or superficiality of anticipatory tools	2 (2.43)
	Facilitator	AF1	The inclusivity of standardisation	2 (2.00)
		AF2	Transparency of the committee’s activities	3 (2.27)
Reflexivity	Motive	RM1	To increase the responsibility of standards	2 (1.82)
		RM2	To increase the market acceptance of standards	2 (2.05)
	Barriers	RB1	A lack of inclusion	2 (2.27)
		RB2	A lack of transparency	3 (3.00)
		RB3	Ambiguous interpretations of the SDO’s rules/guidelines/code of conduct	3 (2.38)
		RB4	‘Hidden’ agendas or goals of committee members	2 (2.14)
		RB5	The complexity of the standardisation process	2 (2.19)
		RB6	The vast number of different standardisation processes	3 (2.71)
		RB7	A lack of impact assessments to reflect on a standard’s impact	2 (2.55)
	Facilitator	RF1	SDO’s regulation and guidelines	2 (2.27)
		RF2	Evaluation tools (e.g. customer feedback forms)	2 (2.19)
		RF3	Training of SDO employees (i.e. consultants)	1 (1.19)
		RF4	Controlling external bodies (e.g. ISO)	3 (2.77)
		RF5	Controlling internal bodies (e.g. policy committees, managers, etc.)	2 (2.41)
		RF6	Technology (e.g. customer platforms)	3 (2.82)
		RF7	External incidents	2 (1.95)
		RF8	A committee’s awareness of its societal obligation	2 (2.05)
Responsiveness	Motive	ResM1	To make a positive societal impact	2 (1.88)
		ResM2	To address the SDGs	3 (2.67)
		ResM3	To create consensus	3 (2.43)
		ResM4	To increase the quality of the standard	2 (1.96)
		ResM5	To increase the market acceptance	2 (2.04)
		ResM6	To maintain the value of a standard	1 (1.43)
	Barrier	ResB1	A lack of clear requirements of when to terminate the process	3 (2.75)
		ResB2	Conflicting goals of committee members	2 (2.26)
		ResB3	‘Hidden’ agendas of committee members	2 (2.05)
		ResB4	The complexity of the standard	2 (1.86)
		ResB5	The involvement of a party in a later stage	2 (2.32)
		ResB6	Reaching consensus becomes harder once a party is emotionally involved	3 (2.52)
		ResB7	Conflicting fundamental values of committee members	2 (2.43)
		ResB8	Some topics are less susceptible to consensus	2 (2.22)
		ResB9	Inclusivity (committee size impedes consensus)	3 (2.36)
		ResB10	Dependencies on other SDOs (e.g. ISO, CEN, etc.)	2 (2.05)
		ResB11	The desire for technological stability (e.g. due to sunk investments)	2 (2.09)
	Facilitator	ResF1	The (partly) online meeting mode	2 (2.18)
		ResF2	A less formal type of standard (e.g. NTA, NPR, NEN-spec)	1 (1.76)
		ResF3	Governmental mandates for standard	2 (1.95)
		ResF4	Societal pressure	1 (1.68)
		ResF5	The severity of the problem at hand	1 (1.48)
		ResF6	The willingness of parties to compromise	1 (1.82)
		ResF7	A committee’s group cohesion	2 (2.41)
		ResF8	Expectations of members (e.g. aligning goals)	2 (1.86)
		ResF9	The frequency of meetings	2 (2.00)
		ResF10	A sense of urgency	2 (1.95)
		ResF11	The context. The national context allows for more control	2 (2.27)
		ResF12	The sector affects the acceptable consensus and improvement speed	2 (2.24)
		ResF13	The mediating skills of chairpersons	2 (1.86)

## Appendix III: An Overview of the most Prevalent Identified Factors

See Table 5

**Table 5:** Overview of factors that were recognised most by consultants

Dimension	Type	No	Factor	Results median ($\overline{{\varvec{x}} }$)
Responsible standardisation	Motive	SM1	The SDO’s obligation to society	1 (1.41)
		SM4	To safeguard the credibility of the SDO	1 (1.63)
Inclusion	Motive	IM1	To increase the responsibility of standards	1 (1.58)
		IM2	To increase the market adoption of standards	1 (1.58)
		IM5	To increase the value of the SDO’s stakeholder network	1 (1.58)
	Barrier	IB1	Unawareness of the process of standardisation	1 (1.62)
		IB10	The cost of involvement	1 (1.76)
Anticipation	Motive	AM1	To increase the responsibility of standards	1 (1.77)
		AM2	To increase the market acceptance of standards	1 (1.78)
		AM4	To prolong the potential relevance of standards	1.5 (2.05)
Reflexivity	Facilitator	RF3	Training of SDO employees (i.e. consultants)	1 (1.19)
Responsiveness	Motive	ResM6	To maintain the value of a standard	1 (1.43)
	Facilitator	ResF2	A less formal type of standard (e.g. NTA, NPR, NEN-spec)	1 (1.76)
		ResF4	Societal pressure	1 (1.68)
		ResF5	The severity of the problem at hand	1 (1.48)
		ResF6	The willingness of parties to compromise	1 (1.82)

## Footnotes

1 By answering: completely agree (1), partly agree (2), neutral (3), partly disagree (4), completely disagree (5), or ‘prefer not to answer’ or ‘don’t know’.
